# Exploring the Ownership of Child-Like Sex Dolls

**DOI:** 10.1007/s10508-022-02422-4

**Published:** 2022-09-19

**Authors:** Craig A. Harper, Rebecca Lievesley

**Affiliations:** NTU Psychology, School of Social Sciences, Nottingham Trent University, 50 Shakespeare Street, Nottingham, NG1 4FQ UK

**Keywords:** Sex dolls, Minor-attracted people, Sexual satisfaction, Sex toys, Sexual abuse, Pedophilia, DSM-5

## Abstract

There is a fervent social debate ongoing that relates to the ownership of child-like sex dolls. On the one hand, some proponents of dolls suggest that they offer a safe sexual outlet for minor-attracted people (MAPs) and could be used in efforts to prevent the sexual abuse of children. On the other side of the debate, child-like dolls are seen as articles that sexualize children, encourage deviant fantasies, and increase offending risk. To date, no empirical analyses have been undertaken with people who own such dolls. In this paper, we present data from child-like sex doll owners (*n* = 85) and MAPs who do not own dolls (*n* = 120) recruited from online forums visited by people who own sex dolls or forums for people with sexual attractions to children. Specifically, we compared their psychological characteristics and proclivities for sexual aggression. Among non-owners, 79.2% of participants declared an interest in owning a sex doll, which is higher than the 20–40% rate reported in adult-attracted samples of non-owners. We found few differences between the groups on most personality variables, with doll owners being less antisocial and anxiously attached than non-owners, but exhibiting more schizotypal traits. Related to offending proclivities, doll ownership was associated with lower levels of sexual preoccupation and self-reported arousal to hypothetical abuse scenarios, but higher levels of sexually objectifying behaviors and anticipated enjoyment of sexual encounters with children. We discuss these data in relation to a functional model of child-like sex doll ownership among MAPs.

**Supplementary Information:**

The online version contains supplementary material available at 10.1007/s10508-022-02422-4.

## Introduction

Among sex research (and broader societal discussions related to sexuality), few topics are currently as polarizing as themes related to sexual attractions to children and the ownership of sex dolls. Indeed, controversies related to the study of minor-attracted people (MAPs) have led to academic dismissals and widespread public backlash (see Asbury, 2021), while state- and national-level legislatures have crafted new laws to restrict the legal ownership of sex dolls in recent years (Prostasia Foundation, 2021).^1^ Although we are beginning to understand that there is a range of both sexual and non-sexual motivations for the ownership of sex dolls among teleiophilic individuals (i.e., those whose sexual attractions are directed toward adults), nothing is currently known about those who own child-like sex dolls (for a review of the literature, see Harper & Lievesley, 2020). Similarly, we do not know anything about the desire for such dolls among those who are sexually attracted to children. In this paper, we present an initial snapshot of the psychological characteristics that are associated with the ownership of child-like sex dolls, and contribute some initial empirical data to ongoing debates about the potential associations between doll ownership and risks related to the sexual abuse of children. In doing so, we sought to answer three over-arching questions:

1. What is the rate of interest in owning child-like sex dolls among MAPs who currently do not have such a doll?
2. Are those who own child-like sex dolls distinguishable from MAPs who do not own such dolls in relation to relevant psychological constructs (e.g., disordered personality traits, attachment difficulties)?
3. Is there any evidence that the owners of child-like sex dolls present an increased risk of sexual offending in comparison with MAPs who do not own dolls?

### What Do We (Not) Know About Child-Like Sex Doll Ownership?

Although the extant literature on the ownership of sex dolls is still in its infancy, the available analyses focus on those who own adult-like dolls (Ferguson, 2014; Harper et al., 2022; Langcaster-James & Bentley, 2018; Su et al., 2019; Valverde, 2012). This means that we know very little about the motivations of those who own child-like dolls, or the effects that such dolls have on psychological states and subsequent behaviors. From a philosophical perspective, Danaher (2019) examined the issue of child-like sex dolls using the lens of legal moralism, which means that scholars cite how the perceived immorality of child-like dolls should act as a basis for their restriction or criminalization, irrespective of the level of objective harm they cause. In doing so, he drew on Strikwerda’s (2017) work to argue that engaging in sexual activity with a child-like sex doll not only encourages and normalizes sexual activity with children, but that it also indicates a lack of moral virtue among the owners of such dolls (see also Danaher, 2017). Relatedly, the ownership of child-like sex dolls has been cited as causing symbolic harm, with Chatterjee (2020) arguing:

‘… permitting a trade in even abstract child sex dolls and robots could be seen as sanctioning and facilitating a public atmosphere that encourages the portrayal of children as sexual objects, and the acceptance and normalization of child abuse’ (p. 34).

Although this is a logical argument to make in light of the evidence related to the conditioning of paraphilic sexual interests (see e.g., Pfaus et al., 2020), it fails to acknowledge the potential for dolls playing a role in the prevention of child sexual abuse. Some professionals have tentatively made this argument (for a summary, see Rutkin, 2016), but it has generally been rejected without much detailed discussion of its validity. In one review of these arguments, the Australian Institute of Criminology (which operates as a branch of the Australian Government) commissioned a report into the effects of child-like sex dolls, specifically in relation to:

1. The promotion of the sexualization of children
2. The extent to doll ownership indicated an escalation from child sexual exploitation material (CSEM) use
3. The association between child-like sex doll ownership and contact sexual offending against children)
4. The use of child-like sex dolls as a tool for the sexual grooming of children.

In the final report, Brown and Shelling (2019) were explicit in their lack of identification of any empirical evidence for any specific hypothesis about the effects of child-like doll ownership (see also Harper & Lievesley, 2020). They concluded that:

‘It is reasonable to assume that interaction with child sex dolls could increase the likelihood of child sexual abuse by desensitizing the doll user to the physical, emotional and psychological harm caused by child sexual abuse and normalizing the behavior in the mind of the abuser. At the same time, there is no evidence of therapeutic benefit from child sex doll use.’ (Brown & Shelling, 2019, p. 8)

As alluded to above, it is plausible that child-like doll ownership is in some way (at least on a theoretical level) associated with proclivities for child sexual abuse. It is equally plausible that doll ownership is associated with child abuse prevention. It is, therefore, concerning that a government-supported agency would seek to promote one side of this debate and downplay the validity of the other, while simultaneously explicitly highlighting the lack of evidence in relation to either hypothesis. Such a conclusion was also reached by Cox-George and Bewley (2018), who purported to assess the implications of the use of sex dolls and robots on patients and professionals in healthcare settings. Again, after their literature search explicitly uncovered no empirical data, they discussed the risks of using dolls in terms of law enforcement interests in dolls, the potential for healthcare providers to be prosecuted for providing illicit materials, and the incitement of public backlash.

### Dolls as Protective, Risky, and/or Functional?

With the above arguments in mind, Harper et al. (2022) set out three potential models of sex doll ownership that are linked to the issue of risk. We outline these below.

#### Model 1: ‘Dolls as Protective’

The first potential model of child-like sex doll ownership purports that dolls could be protective of sexual aggression by offering a safe sexual outlet for those who experience sexual attractions to children. Although the cathartic potential of dolls has never been explored empirically, there is some evidence that access to mainstream pornography is associated with lower rates of sexual violence at the societal level (Ferguson & Hartley, 2009, 2022) which is potentially indicative of people seeking sexual gratification in pornography rather than via the coercion of living victims. Similar trends have been found in relation to child sexual exploitation material, with there being no direct evidence for a link between the consumption of sexually explicit materials depicting children and subsequent engagement in abusive behaviors (Diamond et al., 2011; Seto & Eke, 2005; Seto et al., 2011). Within the context of child-like sex doll ownership, it is possible that being able to own a doll that resembles a preferred sexual target (regarding perceived age or specific physical characteristics) could help some MAPs to achieve sexual satisfaction and to reduce sexual fantasy engagement about real children (Moen & Sterri, 2018; Rutkin, 2016).

#### Model 2: Dolls as Risk-Enhancing

Contrary to the view that child-like sex dolls could reduce the likelihood of owners abusing children, some theorists argue that dolls have the potential to increase owners’ risk levels. At the heart of this view is a tolerance, wherein dolls reinforce sexual attractions to children and, over time leads to a need to engage sexually with real children to achieve the same level of satisfaction. Like the argument that dolls may be protective, this risk-enhancing hypothesis has not been empirically tested but is concluded in various legal and sociological treatises about the legal status of child-like sex doll ownership (e.g., Brown & Shelling, 2019; Chatterjee, 2020; Cox-George & Bewley, 2018; Danaher, 2017, 2019; Strikwerda, 2017).

It is possible that the rehearsal of sexual fantasies and the reinforcement obtained via orgasm could serve to strengthen sexual attractions to children (Arrigo & Purcell, 2001; Pfaus et al., 2020), as well as contributing to the development of implicit theories that are supportive of their sexual abuse (for reviews, see Bartels & Merdian, 2016; Ward & Keenan, 1999). For example, having on-demand sexual access to a sex doll that resembles a child could increase beliefs related to the need for sexual activity when aroused (known as the ‘uncontrollable sex drive’ implicit theory), as well as exaggerated levels of sexual entitlement. Given the advances in AI-enhanced sex robots, there may also be the possibility for perceived consent from some robots resembling children that might increase the perception of owners that children are sexual beings who can give valid consent to sexual activity, with this view being associated with more permissive attitudes about, and a greater proclivity for, offending behavior (Dawson et al., 2009; Mihalides et al., 2004; Ward & Keenan, 1999).

It is in this discussion that we can draw upon theoretical accounts of sexual offending, with Seto’s (2019) motivation-facilitation model being a parsimonious framework for understanding the interactions between these various constructs. Briefly, the model asserts that there are sex-related constructs that may *motivate* offending behavior by directing attention toward preferred sexual outlets. Examples of such constructs include paraphilias (e.g., pedophilia, sexual sadism), blockage (i.e., a lack of prosocial, healthy, and/or consensual sexual outlets), and having a high sex drive. However, there is an acknowledgment that these motivating factors do not fatalistically lead to offending, but instead interact with a variety of *facilitating* factors that increase the likelihood of sexual abuse occurring. At the trait level, issues such as antisociality can increase the likelihood that motivators of offending will be acted upon, while at the state level issues such as intoxication or stress can override normal behavioral controls and lead people to behave in ways that they otherwise would not (Gannon, 2009). In the current context, it may be that ownership of a sex doll strengthens atypical sexual interests or feelings of dissatisfaction with living partners (motivating factors) or contributes to the development of offense-supportive cognitions (facilitating factors).

#### Model 3: Dolls as Functional Life Tools

Away from associations with sexual risk, child-like sex dolls may serve other functions for the people who own them. Of course, there is a primary sexual function for sex dolls in a general sense (Ferguson, 2014; Harper et al., 2022; Langcaster-James & Bentley, 2018; Valverde, 2012), and it is plausible that this masturbatory use of child-like models is also important for MAPs who own dolls. This need not reflect any association (positive or negative) with risks for offending. We also know that men who own sex dolls have non-sexual reasons for doing so, including those that relate to emotional closeness and a lack of viable real intimate partners (Ferguson, 2014; Harper et al., 2022; Su et al., 2019; Valverde, 2012). This may be more pronounced among MAPs who are motivated to own dolls for forming intimate attachments and to provide care for, such as a natural parental instinct in many people (Geary & Flinn, 2001). Given their sexual attractions, it may be difficult for some MAPs to form and maintain intimate relationships with age-appropriate adults, and thus they may miss out on raising children in the kinds of ways that teleiophilic individuals do. Child-like dolls offer them the opportunity to mimic these processes in surrogate parental relationships where sexual motivations for ownership may be secondary. A subsequent potential effect of having a ‘child’ (in doll form) to care for, or to achieve sexual satisfaction with, is an increase in overall life satisfaction and wellbeing. Wellbeing is often a by-product of both life satisfaction (Fergusson et al., 2015) and sexual satisfaction (Carcedo et al., 2020), with all of these being considered primary human goods within the good lives framework (see Ward & Gannon, 2006; Ward & Mars, 2004). As such, dolls need not be positively or negatively related to sexual risk but may instead be seen as a functional part of the lives of individuals who, through their sexual attraction patterns, are unable to live such full lives with real partners or children.

### The Current Study

With these three models in mind, in this exploratory work, we present an initial snapshot of the ownership and use of child-like sex dolls using an anonymous online survey. Specifically, we sought to explore the personality characteristics of such individuals, as well as their levels of sexual risk relative to a non-owner comparison sample of people who are sexually attracted to children. We chose to focus on these kinds of variables in the current study so that we could begin to understand the types of people who own sex dolls from a core personality and individual differences perspective. We omitted measuring constructs such as mental health or psychological distress as these could be a cause or an effect of doll ownership, and thus are better candidates for future work once initial profiles of doll ownership are developed. From the outset, we did not begin with any specific hypotheses or expectations about the kinds of traits or behaviors that might be more prevalent among individuals who own child-like dolls. We are also open to the suggestion that dolls could be risk-enhancing, risk-reducing, or risk-neutral (or indeed that dolls may have a different relation to risk for each individual owner). In this work, we hoped to establish a baseline for the types of constructs that may be relevant to child-like doll ownership, and to inform the sound design of future research efforts in this area.

## Method

### Participants

The data reported here stem from a larger project into the psychology of sex doll ownership with over-arching aims of exploring the psychological characteristics of individuals who own sex dolls, and their relative levels of sexual risk in comparison with non-owner comparison samples. Analyses of men who own adult-like sex dolls have already been published in Harper et al. (2022), with the data reported here referring to those participants who were directed by the survey software into the ‘child-like dolls’ branch of the survey. Eligibility for this branch included the self-declared ownership of at least one child-like sex doll, or self-declared sexual attractions to children. Branching participants in this way enabled us to recruit doll owners and comparison groups using a single anonymous survey link, while still only displaying relevant measures to each subgroup. Women were excluded from the study owing to the gendered nature of doll ownership (Harper & Lievesley, 2020; Langcaster-James & Bentley, 2018; Middleweek, 2021).

To recruit participants, we placed study advertisements on prominent web forums for individuals who own sex dolls, including boards on popular microblogging and social media platforms,^2^ and on two international online forums for individuals with sexual attractions to children seeking peer support. After removing all cases within the dataset with either no data or a negative response to the informed consent question, we were left with a final sample of 205 men who either (a) owned at least one child-like sex doll (*n* = 85), or (b) were attracted to children but did not own any form of sex doll (*n* = 120). The demographic characteristics of each subgroup are given in Table 1.

**Table 1:** Demographic characteristics, by group

Variable	Group	
	Doll owners	Non-owner MAPs
Age (years)	37.8 (± 13.6)	33.7 (± 13.8)
*Country*		
USA	31 (37.3%)	38 (33.9%)
UK and Ireland	4 (4.8%)	14 (12.5%)
Canada	2 (2.4%)	9 (8.0%)
Australia/New Zealand	0 (0.0%)	6 (5.4%)
Mainland Europe	40 (48.2%)	33 (29.4%)
Other	6 (7.2%)	12 (10.7%)
*Relationship status*		
Single (no current relationship)	64 (76.2%)	83 (70.9%)
Married	7 (8.3%)	6 (5.1%)
Divorced	8 (9.5%)	11 (9.4%)
Partnered (unmarried)	5 (6.0%)	17 (14.5%)
*Declared age orientation*		
Adults-only	5 (5.9%)	0 (0.0%)
Children-only	15 (17.9%)	34 (28.3%)
Both (non-exclusive attractions)	64 (76.2%)	86 (71.7%)

Data related to ‘Age’ represent the mean age within each sample, with ± 1 SD in parentheses. All other data represent frequencies, with the percentage equivalent (within the subgroup) in parentheses. Percentages may not always equal 100% due to rounding

### Materials

#### Demographics

We asked participants to provide minimal demographic information to help them to remain anonymous. Basic information as requested, including sex (male/female), age (in years), relationship status (coded into ‘single’, ‘married’, ‘divorced’, and ‘partnered’), country of residence, and sexual orientation for age (with options given to state attractions to adults-only, children-only, or both). No definitions of ‘adult’ and ‘child’ were given in relation to sexual orientation for age.

#### Doll-Related Variables

We asked participants to self-report their doll owner status (yes/no). We did not give a definition of what we meant by ‘doll,’ meaning that different models (e.g., inflatable plastic dolls, synthetic inanimate dolls, AI-incorporated robots) are included in this label. If a participant said that they did own at least one sex doll, they were asked about the type of doll (adult-like/child-like/both), the number of dolls owned, and how long they had owned dolls for. Participants self-rated their doll(s), and we did not give any guidance about what contributed a child-like or adult-like doll. We also asked participants to rate the functions of their dolls as a way to measure how dolls were being used. Using an eleven-point sliding scale anchored from 0 (not at all) to 10 (very much), doll owners rated how much they used their doll(s) for sexual purposes, for non-sexual purposes (e.g., companionship), and for other purposes. The latter option also provided a free-text box for participants to elaborate on these broader doll functions. We also asked how often, in an average month, owners had sex with their doll(s). Non-owners were asked whether they would consider owning one using a binary yes–no response option.

#### Sexuality-Related Variables

We used Snell and Papini’s (1989) Sexuality Scale to measure different facets of participants’ engagement with sexuality. This measure has been widely used in sexuality research to explore sexual self-esteem (e.g., ‘I am confident about myself as a sexual partner’; *α* = 0.91), sexual preoccupation (e.g., ‘I think about sex a great deal of the time’; *α* = 0.89), and sexual depression (e.g., ‘I feel down about my sex life’; *α* = 0.88). Scores obtained using the measure have been found to correlate with self-esteem, sexual self-efficacy, and life satisfaction (see Zimmer-Gembeck & French, 2016), as well as compulsive pornography use (Doornward et al., 2016). There are ten items per subscale, with participants rating each item on a scale ranging from − 2 (strongly disagree), through 0 (neither agree nor disagree), to + 2 (strongly agree). We calculated an average item score for each subscale. We also asked participants how often, in an average month, they had partnered sexual activity.

#### Personality Traits

To examine personality traits exhibited by our participants, we used Hain et al.’s (2016) Personality Styles Inventory, which is a short form of the original 140-item scale produced by Kuhl and Kazén (2009), has subscales for measuring the following DSM-related personality clusters:

- Schizotypal personality (e.g., ‘I often have sudden inspirations’; *α* = 0.56)
- Borderline personality (e.g., ‘My feelings often change abruptly and impulsively’; *α* = 0.53)
- Narcissistic personality (e.g., ‘Being the center of attention really appeals to me’; *α* = 0.47)
- Avoidant personality (e.g., ‘When I feel observed, I become anxious’; *α* = 0.64)
- Obsessive–compulsive personality (‘I am a person with fixed habits’; *α* = 0.57)
- Antisocial personality (e.g., ‘People who want to harm me can count on my retaliation’; *α* = 0.62)

Each cluster is measured using six items that refer to DSM criteria pertaining to each cluster of personality traits, with these being rated using a four-point scale anchored from 1 (does not apply at all) to 4 (fully applies). We calculated an average score for each cluster for each participant. The validity of this measure is supported by data from Kuhl and Kazén’s (2009) original development work, showing correlations in the expected directions between PSDI factor scores and Big 5 personality traits. For example, scores on the borderline personality factor are associated with trait neuroticism (Kuhl & Kazén, 2009), while avoidant personality traits are associated with a fear of making mistakes (Hain et al., 2016). Hain et al. (2016) also found obsessive–compulsive traits to be associated with perfectionism, and narcissistic traits to be associated with increased self-esteem.

#### Emotional Experience

Participants completed Watson et al. (1988) Positive and Negative Affect Scale to provide an index of their experience of emotion. This measure contains a list of 16 different emotions, with participants stating how often they experienced each of these in the preceding week. Ratings for ten positive emotions (e.g., excited, strong, proud) and ten negative emotions (e.g., upset, guilty, ashamed) were collected using a seven-point scale scored from 1 (very slightly or not at all) to 7 (extremely). The measure has been found to correlate highly with mental wellbeing and life satisfaction, which acts as evidence of its construct validity (Crawford & Henry, 2004; Kiesswetter et al., 2020). We first calculated an average score for positive and negative emotions separately, before subtracting the negative score from the positive score. This led to a composite index of emotionality (*α* = 0.85), where positive scores equated to more positive emotional experiences, and negative scores indicated more negative emotional experiences.

#### Attachment Styles

We used Gillath et al. (2009) State Adult Attachment Measure to obtain an indication of participants’ attachment styles. This measure contains subscales that measure traits associated with a secure attachment style (e.g., ‘I feel like I have someone to rely on’; *α* = 0.89) and anxious-insecure attachment style (e.g., ‘I wish someone would tell me they really love me’; *α* = 0.88), and an avoidant-insecure attachment style (e.g., ‘The idea of being emotionally close to someone makes me nervous’; *α* = 0.80). Each subscale contains seven items, which are scored using a seven-point scale anchored from 1 (strongly disagree) to 7 (strongly agree). For each participant, we calculated an average score for each attachment style. This measure has been found to measure stable attachment styles over time (Fraley et al., 2011) and that scores are related to outcomes such as life satisfaction in the expected directions (Stöven et al., 2021).

#### Sexual Objectification

We used the Interpersonal Sexual Objectification Scale—Perpetrator Version (Gervais et al., 2018) to measure the extent to which participants had engaged in sexual objectification during the past 12 months. This is a 15-item measure that asks participants to rate how often in the past year they engaged in a range of behaviors (e.g., ‘Stared at someone’s breasts/chest when you are talking to them’) using a five-point scale anchored from 1 (never) to 5 (almost always). An average score across all items was calculated to provide an index of sexual objectification (*α* = 0.83). Past studies have found this scale to be associated with objectively measured gaze patterns of heterosexual men when they were asked to look at images of female bodies (Baraket et al., 2019), providing support for the validity of this measure.

#### Sexual Offending Proclivity and Past Offending Behaviors

We asked participants to complete the Interest in Child Molestation Scale (Gannon & O’Connor, 2011) to gauge their levels of hypothetical interest in engaging in the sexual abuse of children. This is a measure consisting of five sexual abuse scenarios, which are each followed by three questions pertaining to (1) anticipated levels of sexual arousal, (2) anticipated intention to engage in the stated behavior, and (3) anticipated enjoyment of engaging in the behavior. All questions are rated using a 1–7 scale, with higher scores indicating greater levels of sexual arousal, behavioral intention, and anticipated enjoyment, respectively. We calculated a general index of ‘interest in child molestation’ by averaging all 15 responses (*α* = 0.94), as well as separate indices of ‘arousal’ (*α* = 0.89), ‘behavior’ (*α* = 0.88), and ‘enjoyment’ (*α* = 0.92).

We also asked participants whether they had ever engaged in sexual offending since the age of 18 years. We specifically asked whether participants had had sex with somebody who had not been consenting, whether they had sexual contact with a person below the age of 16 years, and whether they had obtained or viewed sexual images depicting children. As fillers, we also asked about whether participants had acted violently toward another individual or whether they had ever stolen something. In relation to each offending behavior, participants answered either ‘yes,’ ‘no,’ or ‘unsure.’

### Procedure

Advertisements for participation were placed on forums that are known to be frequented by both doll owners and MAPs, with these highlighting the study’s aim of exploring the psychological characteristics of those who own sex dolls. Interested individuals could click on the link which took them to the first page of the survey, and an overview of the project. The survey was hosted on SoSciSurvey to allow for the use of IP-blocking Tor browsers, which are commonly used by MAPs taking part in survey research. Upon providing their consent to take part, participants first completed the demographic and doll-related questions, before completing all other study scales in a randomized order. No questions were mandatory, save for the initial consent procedure. Upon completion of the survey, participants were presented with a comprehensive debriefing screen and links to relevant support services. In line with our institutional ethical approval, all participant data were included if they completed at least one of the study scales. Participants were asked to contact the research team using an anonymous email or letter (citing their unique participant code, which was self-produced after providing their consent) if they wish to withdraw. Specific subsample sizes for individual scales are presented in Table 3.

### Data Analysis Strategy

We conducted our analyses in three stages. In the first stage, we provide some baseline statistics about the ownership of child-like sex dolls, particularly in relation to their functions for owners. Stage two led to us using simple between-groups tests to establish whether on a general level the two groups differed on our measured variables. This involved us running a series of Chi-square and *t* tests. We then ran a binary logistic regression at stage three to explore the prediction of group membership by each of our measured variables while simultaneously controlling for the rest of the data. Given the exploratory nature of our analyses and the independence of each test that was run, we did not make any *p*-adjustments (for a discussion of the nature of *p*-value adjustments in exploratory research, see Rubin, 2017).

## Results

### The Functions of Dolls for Owners

We began by exploring the information provided by child-like doll owners in relation to the functions of their dolls. We specifically asked doll owners to rate the ‘sexual’ and ‘emotional’ functions of their dolls, before giving an option for rating ‘other’ functions along with a free-text input opportunity. We ran a one-way analysis of variance (ANOVA) to examine the differences in function endorsement with the outcome of this not being statistically significant, *F*(2, 142) = 2.75, *p* = .068, *η*^2^_*g*_ = 0.03. This suggests that child-like doll owners are equally using their dolls for sexual reasons (*M* = 6.67, SD = 3.15), emotional reasons (*M* = 6.11, SD = 3.44), and ‘other’ reasons (*M* = 5.12, SD = 3.80). We then looked at the free-text responses accompanying the ‘other’ reasons to look for themes. Prominent other reasons for child-like doll ownership included ‘photography and art’ (*n* = 17; 20% of doll owners), ‘non-sexual intimacy’ (*n* = 17; 20% of doll owners), ‘hobbies’ (*n* = 8; 9.4% of doll owners), ‘non-sexual fantasy play’ (*n* = 4; 4.7% of doll owners), ‘non-intimate care’ (*n* = 2; 2.4% of doll owners), ‘improving quality of life’ (*n* = 2; 2.4% of doll owners), and ‘preventing offending behavior’ (*n* = 1; 1.2% of doll owners).

On average, doll owners had sex with their dolls 8.08 times per month (SD = 9.63; Mdn = 5 times), which was significantly higher than the number of times they engaged in partnered sexual activity (*M* = 1.91 times, SD = 4.97, Mdn = 0 times), *t*(72) = 6.49, *p* < .001, *d* = 0.76. Related to the potential function of dolls as a route to achieving sexual satisfaction among MAPs, we found that 79.2% of our non-owner sample reported an interest in owning a sex doll.

### Between-Groups Analyses

Exploring sexual attraction patterns, we ran a 2 (doll owner vs. comparison sample) × 3 (adult-attracted vs. child-attracted vs. both-attracted) test of association with explore whether doll owners were any more or less likely than non-owners to be exclusively attracted to children. This test was statistically significant, *χ*^2^(2) = 9.54, *p* = .008, Cramer’s *V* = 0.22. In examining the contingency table (Table 2), we saw that exclusive adult-directed attractions were slightly over-represented in the child-like doll owner sample compared to the figure that would be expected by chance. Contrastingly, exclusive attractions to children were slightly more prevalent than expected in the non-owner sample. Non-exclusive attractions were at the levels expected in each group. Acknowledging that some ‘expected’ counts were below five participants (i.e., fewer than five participants in each group were expected to declared adult-only attractions), we also ran Fisher’s Exact Test and confirmed the significance of this analysis (*p* = .007). Removing participants who exclusively declared themselves adult-attracted led to the test being statistically non-significant, *χ*^2^(1) = 2.24, *p* = .134, Cramer’s *V* = 0.13.

**Table 2:** Contingency table for that Chi-square test related to (non-)exclusivity of attraction patterns among child-like doll owners and non-owner MAPs

Attraction Pattern		Group		
		Non-owner	Doll owner	Total
Adult-only	Observed	0 *(2.94)*	5 *(2.06)*	5
Children-only	Observed	34 *(28.82)*	15 *(20.18)*	49
Both (non-exclusive)	Observed	86 *(88.24)*	64 *(61.76)*	150

‘Expected’ counts are presented in italics within parentheses, and reflect the values that would be expected if the observed number of people in each ‘Attraction Pattern’ category were proportionately distributed between the two samples

We then ran a series of between-groups *t* tests to explore differences between the groups on our measured variables.^3^^,^^4^ Descriptive statistics and details of the inferential findings are presented in Table 3. In these tests, we see few differences between child-like doll owners and non-owner MAPs. Where we did see differences, doll owners were significantly less likely (to a moderate-to-large degree) to express a proclivity for sexual abuse in relation to the full-form of the Interest in Child Molestation Scale, or specifically in relation to the questions pertaining to hypothetical arousal and behavioral intentions to engage in child sexual abuse. Doll owners were also less likely to demonstrate anxious attachment styles in comparison to non-owners. Finally, doll owners exhibited more obsessive–compulsive personality traits than non-owners in the comparison group.

**Table 3:** Between-groups differences on measured variables

	Group	*n*	*M*	SD	*M*_diff_ [95% CI]	Inferential test
Partnered sex frequency (per month)	Non-owner	114	1.29	3.78	0.57 [− 0.67, 1.81]	*t*(188) = 0.90, *p* = .367, *d* = 0.13
	Doll owner	76	1.86	4.88		
Interest in child molestation (full)	Non-owner	**41**	**3.00**	**1.50**	− **0.86 [**− **1.49,** − **0.23]**	***t*(77) = ** − **2.71, *p* = .008, *d* = ** − **0.61**
	Doll owner	**38**	**2.14**	**1.31**		
Interest in child molestation (arousal)	Non-Owner	**41**	**3.94**	**1.76**	− **1.32 [**− **2.11, 0.55]**	***t*(77) = ** − **3.39, *p* = .001, *d* = ** − **0.76**
	Doll owner	**38**	**2.61**	**1.72**		
Interest in child molestation (behavior)	Non-owner	**41**	**2.05**	**1.31**	− **0.64 [**− **1.16, 0.11]**	***t*(77) = ** − **2.41, *p* = .018, *d* = ** − **0.54**
	Doll owner	**38**	**1.42**	**1.01**		
Interest in child molestation (enjoyment)	Non-owner	41	3.16	1.80	− 0.79 [− 1.58, − 0.00]	*t*(77) = − 1.99, *p* = .051, *d* = − 0.45
	Doll owner	38	2.37	1.72		
Sexual self-esteem	Non-owner	112	− 0.11	0.88	− 0.01 [− 0.25, 0.26]	*t*(184) = − 0.04, *p* = .970, *d* = − 0.01
	Doll owner	74	− 0.12	0.96		
Sexual preoccupation	Non-owner	113	0.31	0.83	− 0.06 [− 0.32, 0.19]	*t*(187) = − 0.48, *p* = .633, *d* = − 0.07
	Doll owner	76	0.25	0.92		
Sexual depression	Non-owner	115	− 0.08	0.84	− 0.21 [− 0.46, 0.05]	*t*(193) = − 1.56, *p* = .120, *d* = − 0.23
	Doll owner	80	− 0.29	0.98		
Emotionality	Non-owner	118	0.45	1.20	0.22 [− 0.12, 0.56]	*t*(197) = 1.29, *p* = .199, *d* = 0.19
	Doll owner	81	0.67	1.17		
Schizotypal personality	Non-owner	108	2.23	0.50	0.02 [− 0.15, 0.18]	*t*(184) = 0.20, *p* = .839, *d* = 0.03
	Doll owner	78	2.25	0.63		
Borderline personality	Non-owner	108	2.46	0.51	− 0.05 [− 0.21, 0.11]	*t*(184) = − 0.60, *p* = .547, *d* = − 0.09
	Doll owner	78	2.41	0.59		
Narcissistic personality	Non-owner	108	2.29	0.55	0.02 [− 0.14, 0.18]	*t*(184) = 0.20, *p* = .846, *d* = 0.03
	Doll owner	78	2.31	0.53		
Avoidant personality	Non-owner	108	2.21	0.56	0.13 [− 0.04, 0.30]	*t*(184) = 1.49, *p* = .139, *d* = 0.22
	Doll owner	78	2.34	0.60		
Obsessive–compulsive personality	Non-owner	**108**	**2.34**	**0.54**	**0.22 [0.05, 0.38]**	***t*(184) = 2.59, *p* = .010, *d* = 0.39**
	Doll owner	**78**	**2.56**	**0.59**		
Antisocial personality	Non-owner	108	2.14	0.58	− 0.03 [− 0.20, 0.15]	*t*(184) = − 0.29, *p* = .769, *d* = − 0.04
	Doll owner	78	2.11	0.63		
Secure attachment	Non-owner	108	4.47	1.40	− 0.40 [− 0.82, 0.01]	*t*(185) = − 1.90, *p* = .059, *d* = − 0.28
	Doll owner	79	4.07	1.49		
Anxious-insecure attachment	Non-owner	**108**	**4.82**	**1.32**	− **0.67 [**− **1.07,** − **0.26]**	***t*(185) = ** − **3.24, *p* = .001, *d* = ** − **0.48**
	Doll owner	**79**	**4.15**	**1.48**		
Avoidant-insecure attachment	Non-owner	108	3.56	1.26	0.16 [− 0.21, 0.52]	*t*(185) = 0.83, *p* = .406, *d* = 0.12
	Doll owner	79	3.71	1.24		
Sexual objectification	Non-owner	104	1.67	0.45	0.09 [− 0.07, 0.24]	*t*(178) = 1.12, *p* = .263, *d* = 0.17
	Doll owner	76	1.75	0.57		

Variables with scores that differ significantly between the groups are presented in bold typeface

We finished this first stage of analysis by looking at the relative levels of self-reported sexual offending among both samples, comparing the proportions of child-like doll owners and non-owner comparisons that reported engaging in non-consensual sexual activities with others, having sexual activity with a child, and obtaining or viewing indecent images of children. We ran a 2 (doll owner vs. comparison sample) × 3 (‘yes,’ ‘no,’ or ‘unsure’ in relation to past offending behaviors)^5^ test of association separately for each offense type. None of these tests were statistically significant, indicating similar proportions of self-reported offending in both samples (see Table 4):

- *Non-consensual sex* *χ*^2^(2) = 1.95, *p* = .376, Cramer’s *V* = 0.11 (Fisher’s Exact Test *p* = .377)
- *Sexual activity with a child* *χ*^2^(2) = 0.66, *p* = .720, Cramer’s *V* = 0.06 (Fisher’s Exact Test *p* = .760)
- *Obtaining or viewing indecent images of children* χ^2^(2) = 3.34, *p* = .188, Cramer’s *V* = 0.14 (Fisher’s Exact Test *p* = .189)

**Table 4:** Contingency table for the Chi-square test related to relative proportions of self-reported sexual offending among child-like doll owners and non-owner MAPs

Offending	Group		
	Non-owner	Doll owner	Total
*Non-consensual sex*			
No			
Observed	86	61	147
Expected	84.84	62.16	147
Yes			
Observed	8	10	18
Expected	10.39	7.61	18
Unsure			
Observed	7	3	10
Expected	5.77	4.23	10
*Sexual activity with a child*			
No			
Observed	81	56	137
Expected	79.4	57.6	137
Yes			
Observed	16	15	31
Expected	17.97	13.03	31
Unsure			
Observed	5	3	8
Expected	4.64	3.36	8
*Obtaining or using indecent images of children*			
No			
Observed	28	27	55
Expected	31.74	23.26	55
Yes			
Observed	63	36	99
Expected	57.14	41.86	99
Unsure			
Observed	10	11	21
Expected	12.12	8.88	21

‘Expected’ counts reflect the values that would be expected if the observed number of people in each ‘Offending’ category were proportionately distributed between the two samples

### Predicting ‘Doll Owner’ Group Membership

To explore how our measured variables could distinguish between child-like doll owners and non-owning MAPs, we ran a binary logistic regression using age, exclusivity of attractions to children, and all our measured variables as predictors.^6^ The model was statistically significant and correctly classified 90.6% of participants into their correct group, Nagelkerke’s pseudo-*R*^2^ = 0.79, χ^2^(20) = 57.80, *p* < .001.

Exploring the predictors of group membership (Table 5), we see that doll owners were significantly older than non-owners. They were also more likely to exhibit schizotypal personality traits, to report engaging in sexually objectifying behaviors than non-owners, and reported more anticipated enjoyment in relation to hypothetical child sexual abuse scenarios. In contrast, they were significantly less sexually preoccupied than non-owners, less antisocial, and demonstrated a lower level of self-reported arousal to child sexual abuse scenarios. Doll owners were also less anxiously attached than non-owners.

**Table 5:** Binary logistic regression distinguishing doll owners from non-owner MAPs

Predictor	B	SE	*Z*	*p*	OR
Intercept	− 13.04	8.91	− 1.46	.143	2.17E−06
Age	**0.12**	**0.06**	**1.99**	**.047**	**1.12**
Exclusivity of minor attraction	− 3.11	1.79	− 1.74	.083	0.04
Partner sex frequency (per month)	0.04	0.12	0.29	.770	1.04
Interest in child molestation (arousal)	− **4.63**	**1.98**	− **2.33**	**.020**	**0.01**
Interest in child molestation (behavior)	− 2.65	1.46	− 1.82	.070	0.07
Interest in child molestation (enjoyment)	**4.15**	**1.89**	**2.19**	**.028**	**63.46**
Sexual self-esteem	0.21	1.01	0.21	.835	1.23
Sexual preoccupation	− **3.93**	**1.87**	− **2.11**	**.035**	**0.02**
Sexual depression	− 0.12	1.19	− 0.10	.921	0.89
Emotionality	2.53	1.52	1.67	.095	12.55
Schizotypal personality	**5.14**	**2.37**	**2.17**	**.030**	**171.51**
Borderline personality	0.87	1.27	0.68	.494	2.38
Narcissistic personality	− 2.11	1.55	− 1.37	.171	0.12
Avoidant personality	9.08	4.83	1.88	.060	8790.17
Obsessive–compulsive Personality	− 0.93	1.97	− 0.47	.637	0.39
Antisocial personality	− **8.07**	**3.84**	− **2.10**	**.036**	**3.14E-04**
Secure attachment	− 0.25	0.84	− 0.29	.768	0.78
Anxious-insecure attachment	− **2.43**	**1.18**	− **2.06**	**.039**	**0.09**
Avoidant-insecure attachment	0.14	0.71	0.20	.843	1.15
Sexual objectification	**8.86**	**4.29**	**2.07**	**.039**	**7034.46**

*B* estimates represent the log odds of belonging to the ‘doll owner’ group*.* ‘Exclusivity’ variable coded as 0 = non-exclusively attracted to children, 1 = exclusively attracted to children. Significant predictors of group membership are presented in bold typeface. We have provided scientific notation for odds ratios less than 0.00 to facilitate future meta-analyses

### Exploring the Potential Doll Ownership-Abuse Relationship

We next ran a series of moderation analyses to explore whether there was support for a model akin to Seto’s (2019) motivation-facilitation model in our data.^7^ Here, it was not our goal to directly suggest that sex doll ownership is directly applicable to this theoretical framework. Instead, Seto (2019) talks about the interactivity of variables in sexual offending, highlighting that this form of offending behavior is not explained easily by single-factor models. In line with this idea, we sought to investigate whether sex doll ownership moderated the effect of relevant constructs to this model and a proclivity for sexual offending. In constructing our models, we selected the behavior items of the Interest in Child Molestation Scale as the outcome, as there is debate about whether self-reported arousal and anticipated enjoyment actually constitute an offending risk in MAP samples (see the Limitations and Future Directions section below for a discussion of this). Sex doll ownership status was added as a moderator to explore whether owning a doll exaggerated (or mitigated) the predictive effects of variables that Seto (2019) identified as being associated with sexual offending, or which were expressed differently between-groups in our previous analyses. The predictor variables we chose were:

- Sexual preoccupation (as an indicator of sex drive)
- Frequency of partnered sexual activity (as an indicator of high sex drive)
- Sexual objectification (as an indicator of intense mating effort)
- Antisocial personality traits (due to its presence as a facilitating factor in the motivation-facilitation model)
- Obsessive–compulsive personality traits (due to the significant between-groups difference in these traits, reported in Table 3)
- Anxious-insecure attachment style (due to the significant between-groups difference in these traits, reported in Table 3)

In constructing the models in this configuration, we were able to test whether the effects of the above variables on proclivities for sexual abuse were different among sex doll owners than among MAPs who did not own dolls. Model coefficients are presented in Table 6.

**Table 6:** Coefficients for moderation models predicting behavioral proclivities for sexual abuse

	Predictor					
	Sexual preoccupation	Partnered sex frequency	Sexual objectification	Antisocial personality	Obsessive–compulsive personality	Anxious-insecure attachment
Predictor	− 0.14	− 0.03	− 0.22	0.01	− 0.12	0.17
Doll ownership	− 0.63*	− 0.58*	− 0.65*	− 0.67*	− 0.77**	− 0.52
Predictor × doll ownership	− 0.15	0.06	0.43	− 0.33	0.67*	− 0.31

Figures represent unstandardized *B* values

*p < .05

**p < .01

As reported in Table 6, in all of our models we found the predictors to not be related to behavioral proclivities for child sexual abuse. However, we found sex doll ownership to be associated with lower proclivities (consistent with the previous analysis). Exploring moderation, we only found a significant effect in the model pertaining to obsessive–compulsive personality traits. When breaking this interaction down into its simple main effects, we found a non-significant positive relationship between obsessive–compulsive traits and abuse proclivities among doll owners (*B* = 0.37, 95% CI [− 0.26, 0.10], *p* = .249) and a non-significant negative relationship among those who did not own dolls (*B* = − 0.50, 95% CI [− 1.10, 0.10], *p* = .102). As such, it appears that the opposite directions of the estimates of these main effects, despite their non-significant nature, is what contributed to the statistically significant interaction.

## Discussion

In this work, we have been able to present what we believe to be the first empirical analysis of child-like sex doll ownership. In doing so, we can begin to build a picture of who child-like doll owners are, their motivations for doll ownership, and the potential associations between such doll ownership and proclivities for the sexual abuse of children. This is of pivotal timing against a backdrop of philosophical and legal theorizing about the dangers of child-like doll ownership within an evidential vacuum (Brown & Shelling, 2019; Chatterjee, 2020; Cox-George & Bewley, 2018; Danaher, 2017, 2019; Strikwerda, 2017).

## Summary of Key Results

In relation to owners’ stated functions of child-like dolls, we found no evidence that sexual reasons were more highly endorsed than more emotional functions of dolls (e.g., dolls being used as a form of emotional intimacy or companionship). Other reasons for doll ownership included photography, non-sexual intimacy (e.g., for companionship), and non-sexual fantasy play, suggesting that the ownership of child-like dolls is far from a simplistic issue that can be explained by appealing to arguments related to sexual deviance. Among minor-attracted non-owners, we observed interest in owning a child-like doll from 79.2% of participants, which is much higher than the observed rate of doll interest of around 20–40% in teleiophilic populations (see Harper et al., 2022; Knox et al., 2017; Szczuka & Krämer, 2016). We acknowledge that true prevalence rates of interest in owning a sex doll would require a larger and more representative sample of MAPs than we have in the current study. However, this estimate indicates that a relatively large proportion of MAPs may have some interest in owning a doll. This is perhaps unsurprising, though, given the nature of sexual attractions to children and the natural striving for some degree of sexual satisfaction. Within this context, it is also unsurprising that doll owners engaged in sexual activity with their dolls significantly more often than they engaged in partnered sexual activity. This is concordant with analyses of adult-like sex doll owners, who report engaging in sexual activity with their doll more often than having sex with a living partner (Harper et al., 2022), and with the broader sexological literature that suggests solitary masturbation is more frequent than partnered sexual activity (Herbenick et al., 2021). Although the issue of engaging more with a doll than a living partner is of interest from an interpersonal perspective, it should be highlighted that engaging sexually with a doll is fundamentally an act of masturbation. As such, comparisons between doll owners’ sexual activities with dolls, and non-owners’ solitary masturbation rates, might serve as a better indicator of whether the ownership of a sex doll leads to unexpectedly high levels of sexual activity in a broader sense.

In contemporary discourse around doll ownership, there is a tendency to frame doll sex ownership through a risk-based lens (Brown & Shelling, 2019; Cox-George & Bewley, 2018; Danaher, 2017, 2019). Within these discussions there is an implicit (and sometime explicit) assumption that the ownership of sex dolls—particularly dolls that resemble children—is inherently risky and associated with a proclivity for sexual offending. We found no such evidence of this within our data, with the behavioral proclivity and arousal to hypothetically engaging in child sexual abuse being lower among child-like sex doll owners than among MAPs who did not own a sex doll. This was even the case after controlling for their comparatively higher relative self-reported anticipated enjoyment of sexual engagement with children, and their higher rates of engaging in objectifying behaviors. As such, some of the concerns about child-like doll owners being at a greater risk of engaging in the sexual abuse of children might be misplaced. Indeed, when asking participants directly about their engagement with criminal activities, we found that there were no differences in self-reported offending between those who owned child-like dolls and those who did not.

From a personality perspective, we found that child-like doll owners exhibited more schizotypal traits, but were less antisocial, than MAPs who did not own such dolls. Thinking about the traits of these two forms of personality this is perhaps unsurprising when considering the intimate relationships that sex doll owners commonly report having with their dolls (Langcaster-James & Bentley, 2018; Valverde, 2012) That is, schizotypal personality traits are associated with social withdrawal and creative thinking (Moorman & Samuel, 2018; Yasuyama et al., 2017), whereas antisocial traits are associated with disinhibition and interpersonal detachedness (Benning et al., 2003; Venables et al., 2014). Within this context, it is understandable why higher levels of schizotypal traits and lower levels of antisocial trait might be more common personality profiles in doll owners, who appear to have more difficulties in forming relationships with real partners and fill this void with an emotional bond with their dolls (Lievesley et al., 2022). Although this could be questioned in the current sample owing to the minor-attracted nature of most participants, it should be noted that the doll owners in this work were no more likely to be exclusively attracted to children than those who did not own a doll, and so it is likely that any potential surrogacy of real relationships with bonds with dolls is better explained by their personality characteristics than by their sexual attraction patterns. We also found that doll owners were less likely to exhibit anxious attachment styles, suggesting a lesser need to form bonds with individual partners, and a reduced emphasis on the achievement of reassurance from others. Among doll owners this likely stems from an acknowledgment that they have a marginalized intimate lives and face disapproval and stigma from other. As such, interpersonal detachment might serve to protect doll owners from upset in everyday life, even if this means detachment from possible future intimate relationships.

When conducting an exploratory analysis of moderating effects of doll ownership on relationships between theoretically supported motivators of abuse and self-reported offending proclivities, we again found very little. This may be reflective of a lack of applicability of Seto’s (2019) motivation-facilitation model to the sex doll ownership context. However, it is important to note that the sample size was relatively small, meaning that statistical power for testing interaction effects is likely to be low. There was also a low average score on the behavioral proclivity items of the Interest in Child Molestation Scale (Gannon & O’Connor, 2011), meaning that there may not have been enough variance in the outcome variable for the models to explain.

### A Functional Model of Child-Like Doll Ownership?

The findings presented here can begin to help us to link child-like doll ownership effects to the three models outlined in Harper et al. (2022). Central to at least two of these models is the association between doll ownership and risks of committing acts of sexual abuse, while the third model adopts a more interactive approach to motivations for, and effects of, doll ownership.

The ‘dolls as protective’ model would hypothesize that doll ownership would be associated with a lower level of sexual risk when compared to an appropriate non-owner comparison group. Although self-reported arousal levels (both in relation to responses to hypothetical child sexual abuse scenarios, and general levels of sexual preoccupation) were lower among doll owners than non-owning MAPs, we found no differences between the groups in self-reported behavioral intentions to engage in child molestation, or in self-reported past offending behaviors. As such, we did not find specific evidence for the ‘dolls as protective’ model.

According to the ‘dolls as risky’ model, we would expect those who own child-like sex dolls to demonstrate an increased proclivity for sexual offending. Although we found that doll owners engaged in more objectifying behaviors (e.g., staring at someone’s body or evaluating their physical appearance) and reported a greater level of hypothetical enjoyment in abusing a child (as compared to MAPs who did not own dolls), we found no evidence of an increased proclivity to engage in sexual abuse. This was the case when considering both hypothetical behavioral intentions (as measured via the Interest in Child Molestation Scale; Gannon & O’Connor, 2011) and self-reported past offending behaviors. As such, we did not find evidence for this risk-enhancing model of doll ownership.

The final model of doll ownership sees the motivations and effects of child-like sex dolls as functional and suggests that those who own such dolls do so because of some functional need that they have. As such, if dolls are to be seen as functional then their ownership will likely be unrelated to abuse proclivities at the group level (as reported above), but may be correlated with specific psychosocial or personality constructs. We did find evidence for this, with doll owners being less likely to have anxious attachment styles, which is indicative of a reduced need to be attached to intimate partners, and in some cases a desire to be relatively independent. At the group difference level, we also found that child-like doll owners exhibited more obsessive–compulsive personality traits than non-owners. This might indicate a need for order that is achievable by forming relationships with dolls (who are predictable and controllable) but not with living partners (who can be less predictable and controllable). Although this finding might be considered provisional in light of the poor psychometric outcomes associated with the Personality Styles Inventory (Hain et al., 2016), we believe that the ownership of child-like sex dolls may be explained by the functional roles (e.g., attachment formation, control over one’s environment, safe sexual outlets related to preferred sexual targets) that such dolls can serve for some MAPs.

### Limitations and Future Directions

One of the clearest limitations of the current study is its reliance on the self-report method of data collection. Although most psychological research uses this approach, topics such as child-like sex doll ownership are particularly susceptible to self-presentation biases in the context of ongoing legislative discussions about their criminalization (see Prostasia Foundation, 2021). We attempted to overcome a motivation for socially desirable responding by not tracking IP addresses of our participants, and by using SoSciSurvey, which is a survey platform that allows users to access online questionnaires using Tor browsers. Despite this safeguard, it is still possible that some participants may have responded in such a way to avoid increased perceptions of the risks posed by those who own child-like sex dolls. Future work in this area might look to include impression management measures to quantify this susceptibility to ‘faking good.’

The data reported in this paper are cross-sectional and correlational, which inherently means that we cannot make causal arguments about the directions of the relationships reported earlier. Our observation that rates of arousal to child abuse scenarios were lower among doll owners, and that anticipated enjoyment of such hypothetical abuse was higher, might be inferred as doll ownership explaining such differences. Finding that child-like doll owners engage in more objectifying behaviors might be perceived as being the result of their owning of dolls. The finding that those who own dolls are less likely than non-owners to be sexually preoccupied might be suggested as the result of dolls satiating the sexual urges of their owners, but the directional nature of these relationships is only able to be fully demonstrated using longitudinal designs that adopt a prospective approach. This is, researchers should collect baseline data in relation to these types of variables and to re-collect such data at regular timepoints following a doll’s initial purchase. In doing this, it would be possible to identify whether those who own dolls differ from non-owners in important ways even before they own a doll, or whether such differences emerge following (and, as such, because of) doll ownership. There are also substantial caveats to mention in relation to the self-selecting nature of the sample. That is, we (necessarily) used a convenience sampling approach to survey MAPs rom online forums about their doll ownership (or lack thereof). Given the heavy stigma surrounding child-like doll ownership there is the potential for bias not only in responses, but also in interest and willingness to take part in such research. Future work might look to recruit people to studies at the point of purchase, though this is likely to need considerable planning considering the usually illegal nature of purchasing dolls that are explicitly labeled as child-like.

One potential issue with our measurement of risk was the use of the Interest in Child Molestation Scale (Gannon & O’Connor, 2011). Principally, no work exists (to our knowledge) that links scores on this scale to actual offending behavior, and instead this acts as a proxy for potential interests in sexual offending against children. Going further, and interpreting our data in light of recent theoretical advances in the understanding of sexual offending, it is clear that arousal is not automatically akin to risk (see e.g., Seto, 2019). As such, using self-reported ‘arousal’ (and even ‘enjoyment’) in relation to a hypothetical sexual encounter between an adult and a child may be a misleading indicator of risk in MAP samples. That is, if we asked teleiophilic individuals about their levels of arousal and anticipated enjoyment of a sexual encounter with another adult, high scores would be consistent with their stated sexual attraction patterns, rather than be an indicator of sexual aggression risk (unless non-consensual aggression was a part of the scenarios used). As such, although hypothetical measures such as the Interest in Child Molestation Scale are currently the field’s best attempt to estimate abuse proclivity in MAP samples, more work in needed to develop accurate assessments of sexual abuse risk in non-offending populations living in the community.

Relatedly to this we did not ask participants about the type of dolls that they owned, which means that we cannot account for any differences that may be present between those who own simplistic inflatable dolls, inanimate silicone models, or dolls with embedded artificial intelligence. It is certainly plausible that each of these categories of doll are associated with different psychological profiles in relation to their owners, especially as people move away from purely sexual motivations and consider their dolls to be more akin to an intimate partner. Future work should look to explore this possibility. Similarly, the perceived ‘age’ of child-like dolls should be considered. That is, there may be observable differences in the effects of particularly young-looking dolls (i.e., those which may align with a pedophilic sexual attraction pattern) than of dolls that resemble teenagers. We might expect those with particular chronophilic attraction patterns to interact with dolls that best resemble their age of attraction, but this possibility was not explored in the current work.

Our omission of definitions about what we meant by ‘adult’ and ‘child’ (both in relation to attractions to others, and to the ages of any dolls owned) could also be seen as a limitation. By way of example, this lack of definitional clarity means that someone could accurately describe themselves as being attracted to both adults and children because they find older teenagers (e.g., 16- or 17-year-olds) attractive, even if they are not attracted to children that would meet our criteria for ‘minor attraction (i.e., under 16 s). Similarly, doll owners might have reported having a child-like doll because they have a doll that looks like a teenager (perhaps a doll with smaller breasts, or dressed in such a way that suggests youth). When thinking about lay definitions of ‘child,’ it is fair to suggest that many may infer younger ages, despite our meaning being below the age of 16 years, and the United Nations’ legal definition of 18 years. Future work should seek to clarify what participants consider to be a ‘child,’ and offer definitions or guidance about what researchers mean when using such terms.

In light of a possible functional use of child-like sex dolls, it might also be a useful direction for future research to explore the controlled use of such dolls among MAPs in therapeutic contexts. In this work, we have not found any evidence that those who own child-like dolls are at an increased risk for sexual offending against children. We are also aware that MAPs struggle to achieve sexual satisfaction due to their attraction patterns and both the morality and legality of sexual contact with children (Mundy & Cioe, 2019). Among non-owners in our sample, 79.2% reported an interest in owning a doll. This suggests a potentially large pool of individuals who could gain from the functional benefits of dolls (e.g., the achievement of sexual satisfaction and the wellbeing benefits associated with this; Carcedo et al., 2020; Ward & Gannon, 2006) without an increase in child abuse risk. Of course, there is also a possibility that having such a sexual outlet could reduce sexual preoccupation among MAPs who are currently unable to achieve sexual satisfaction, and that such a reduction could reduce motivations for sexual offending (for a theoretical model, see Seto, 2019). The hypothetical use of child-like dolls in sexual abuse prevention contexts has been raised previously (see Harper & Lievesley, 2020; Rutkin, 2016), and future research might explore this possibility in well-controlled studies that embed ongoing measures of both risk and wellbeing to track the range of functional outcomes associated with doll ownership.

### Conclusions

In this study, we have presented what we believe to be the first empirical study into the psychological and risk-related constructs associated with the ownership of child-like sex dolls. In contrast to moralistic publications citing the potential risks of such dolls in related to child sexual abuse (Chatterjee, 2020; Danaher, 2017, 2019; Strikwerda, 2017), we found evidence for their functional use. In light of this, we hope that those working in psychological and sex science can launch systematic studies that explore how to best support MAPs in their search for safe sexual outlets, with ultimate aims of improving levels of mental health in this population and, subsequent to this, the prevention of the sexual abuse of children.

## Supplementary Information

Below is the link to the electronic supplementary material.

Supplementary file1 (DOCX 49 KB)

## Data Availability

To preserve the anonymity and confidentiality of those taking part in this exploratory work, the data related to this research is not publicly available.

## Footnotes

1 We define “minor attraction” according to past literature that treats attractions to people below the age of 16 (known by the label *ephebophilia*) as the cut-off. We are aware that there is debate about the suitability of this label, including where to place cut-offs for ages of attraction (particularly in light of United Nations designations of 18 years being the threshold for adulthood. However, we use “MAP” in this way to be in keeping with the emergent literature in this area, and to acknowledge the variable age of consent across the world (where the age of 16 is most commonly used, including in the countries of those represented in our sample).

2 To protect participants’ anonymity in this research project, we assured them that we would not reveal the specific names of the forums and websites that were used for recruitment. None of the MAP-related forums that we used to recruit participants take an explicit position on the ownership of sex dolls, though one does have an association with a charitable organization that has called for increased research into sex dolls before legislation is formed.

3 An anonymous reviewer asked us to re-run these analyses with participants’ age as a covariate. We did this and found the pattern of results to be very similar. The only differences were that behavioral proclivity (*p* = .061) and obsessive–compulsive personality traits (*p* = .072) became statistically non-significant, while enjoyment proclivity became statistically significant (*p* = .049). Age was unrelated to any of the variables. A table summarizing these results is available in the Supplementary Materials accompanying this article online.

4 A second anonymous reviewer asked us to re-run our analyses after removing those participants who only self-declared sexual attractions to adults. After doing this the pattern of results held, except for enjoyment proclivity becoming statistically significant (*p* = .017) and obsessive–compulsive personality traits becoming statistically non-significant (*p* = .080). All effect sizes were comparable to the full sample analysis. A table of these revised results is available in the Supplementary Materials accompanying this article online.

5 At the request of the handling editor, we also re-ran this analysis by grouping responses to ‘yes’ and ‘unsure’ into a single response. Grouping the data like this did not alter the results. Contingency tables are presented in the Supplementary Materials accompanying this article online.

6 At the request of an anonymous reviewer, we re-ran this analysis after excluding those who only declared a sexual attraction to adults. The general pattern of results held, except for the effect of age becoming statistically non-significant (*p* = .051). A table of these revised results is available in the Supplementary Materials accompanying this article online.

7 This was not an explicit aim at the outset of this study, but was suggested as a useful exploration by an anonymous peer-reviewer.
