# Supplementary material for: Exploring the Ownership of Child-Like Sex Dolls
Source: Arch Sex Behav. 2022 Sep 19;51(8):4141–56. doi: 10.1007/s10508-022-02422-4 (PMC9663384; doi:10.1007/s10508-022-02422-4)
Supplement: Supplementary file 1 — Supplementary file1 (DOCX 49 KB) [file 10508_2022_2422_MOESM1_ESM.docx]

| **Table S1:** Between-groups analysis (with age as a covariate) | | | | | | | | | | | | | |
| --- | --- | --- | --- | --- | --- | --- | --- | --- | --- | --- | --- | --- | --- |
|  |  |  |  |  |  |  |  |  |  |  |  |  |  |
|  | | **Dependent Variable** | | **Sum of Squares** | | **df** | | **Mean Square** | | ***F*** | | ***p*** | |
| Group_MAP_Doll |  | SexFreq_Partner |  | 30.330 |  | 1 |  | 30.330 |  | 1.130 |  | 0.292 |  |
|  |  | **Proc_T** |  | **12.297** |  | **1** |  | **12.297** |  | **5.846** |  | **0.019** |  |
|  |  | **Proc_A** |  | **33.167** |  | **1** |  | **33.167** |  | **10.866** |  | **0.002** |  |
|  |  | Proc_B |  | 5.449 |  | 1 |  | 5.449 |  | 3.632 |  | 0.061 |  |
|  |  | **Proc_E** |  | **12.586** |  | **1** |  | **12.586** |  | **4.017** |  | **0.049** |  |
|  |  | Sex_Esteem |  | 0.006 |  | 1 |  | 0.006 |  | 0.007 |  | 0.932 |  |
|  |  | Sex_Preocc |  | 0.066 |  | 1 |  | 0.066 |  | 0.078 |  | 0.782 |  |
|  |  | Sex_Dep |  | 0.146 |  | 1 |  | 0.146 |  | 0.214 |  | 0.645 |  |
|  |  | Emo_Avg |  | 0.879 |  | 1 |  | 0.879 |  | 0.695 |  | 0.408 |  |
|  |  | Schiz_Avg |  | 0.344 |  | 1 |  | 0.344 |  | 0.818 |  | 0.369 |  |
|  |  | Bord_Avg |  | 0.002 |  | 1 |  | 0.002 |  | 0.006 |  | 0.939 |  |
|  |  | NarPD_Avg |  | 0.100 |  | 1 |  | 0.100 |  | 0.285 |  | 0.596 |  |
|  |  | AvPD_Avg |  | 1.114 |  | 1 |  | 1.114 |  | 3.356 |  | 0.072 |  |
|  |  | OCPD_Avg |  | 1.206 |  | 1 |  | 1.206 |  | 3.217 |  | 0.078 |  |
|  |  | Antisoc_Avg |  | 0.041 |  | 1 |  | 0.041 |  | 0.104 |  | 0.748 |  |
|  |  | SecAtt_Avg |  | 3.244 |  | 1 |  | 3.244 |  | 1.664 |  | 0.202 |  |
|  |  | **AnxAtt_Avg** |  | **14.697** |  | **1** |  | **14.697** |  | **10.119** |  | **0.002** |  |
|  |  | AvoAtt_Avg |  | 0.577 |  | 1 |  | 0.577 |  | 0.412 |  | 0.523 |  |
|  |  | Obj_Avg |  | 0.391 |  | 1 |  | 0.391 |  | 1.468 |  | 0.230 |  |
| Age |  | SexFreq_Partner |  | 10.417 |  | 1 |  | 10.417 |  | 0.388 |  | 0.536 |  |
|  |  | Proc_T |  | 0.268 |  | 1 |  | 0.268 |  | 0.127 |  | 0.722 |  |
|  |  | Proc_A |  | 1.066 |  | 1 |  | 1.066 |  | 0.349 |  | 0.557 |  |
|  |  | Proc_B |  | 4.323 |  | 1 |  | 4.323 |  | 2.881 |  | 0.095 |  |
|  |  | Proc_E |  | 0.004 |  | 1 |  | 0.004 |  | 0.001 |  | 0.970 |  |
|  |  | Sex_Esteem |  | 1.996 |  | 1 |  | 1.996 |  | 2.367 |  | 0.129 |  |
|  |  | Sex_Preocc |  | 1.620 |  | 1 |  | 1.620 |  | 1.905 |  | 0.172 |  |
|  |  | Sex_Dep |  | 0.752 |  | 1 |  | 0.752 |  | 1.104 |  | 0.297 |  |
|  |  | Emo_Avg |  | 0.000 |  | 1 |  | 0.000 |  | 0.000 |  | 0.997 |  |
|  |  | Schiz_Avg |  | 0.271 |  | 1 |  | 0.271 |  | 0.644 |  | 0.425 |  |
|  |  | Bord_Avg |  | 3.952 |  | 1 |  | 3.952 |  | 9.515 |  | 0.003 |  |
|  |  | NarPD_Avg |  | 0.003 |  | 1 |  | 0.003 |  | 0.010 |  | 0.922 |  |
|  |  | AvPD_Avg |  | 1.251 |  | 1 |  | 1.251 |  | 3.768 |  | 0.057 |  |
|  |  | OCPD_Avg |  | 0.839 |  | 1 |  | 0.839 |  | 2.240 |  | 0.140 |  |
|  |  | Antisoc_Avg |  | 0.063 |  | 1 |  | 0.063 |  | 0.158 |  | 0.692 |  |
|  |  | SecAtt_Avg |  | 7.423 |  | 1 |  | 7.423 |  | 3.808 |  | 0.056 |  |
|  |  | AnxAtt_Avg |  | 0.104 |  | 1 |  | 0.104 |  | 0.072 |  | 0.790 |  |
|  |  | AvoAtt_Avg |  | 2.746 |  | 1 |  | 2.746 |  | 1.965 |  | 0.166 |  |
|  |  | Obj_Avg |  | 0.023 |  | 1 |  | 0.023 |  | 0.085 |  | 0.772 |  |
| Residuals |  | SexFreq_Partner |  | 1663.737 |  | 62 |  | 26.834 |  |  |  |  |  |
|  |  | Proc_T |  | 130.408 |  | 62 |  | 2.103 |  |  |  |  |  |
|  |  | Proc_A |  | 189.245 |  | 62 |  | 3.052 |  |  |  |  |  |
|  |  | Proc_B |  | 93.028 |  | 62 |  | 1.500 |  |  |  |  |  |
|  |  | Proc_E |  | 194.244 |  | 62 |  | 3.133 |  |  |  |  |  |
|  |  | Sex_Esteem |  | 52.293 |  | 62 |  | 0.843 |  |  |  |  |  |
|  |  | Sex_Preocc |  | 52.697 |  | 62 |  | 0.850 |  |  |  |  |  |
|  |  | Sex_Dep |  | 42.199 |  | 62 |  | 0.681 |  |  |  |  |  |
|  |  | Emo_Avg |  | 78.452 |  | 62 |  | 1.265 |  |  |  |  |  |
|  |  | Schiz_Avg |  | 26.109 |  | 62 |  | 0.421 |  |  |  |  |  |
|  |  | Bord_Avg |  | 25.748 |  | 62 |  | 0.415 |  |  |  |  |  |
|  |  | NarPD_Avg |  | 21.790 |  | 62 |  | 0.351 |  |  |  |  |  |
|  |  | AvPD_Avg |  | 20.587 |  | 62 |  | 0.332 |  |  |  |  |  |
|  |  | OCPD_Avg |  | 23.233 |  | 62 |  | 0.375 |  |  |  |  |  |
|  |  | Antisoc_Avg |  | 24.556 |  | 62 |  | 0.396 |  |  |  |  |  |
|  |  | SecAtt_Avg |  | 120.840 |  | 62 |  | 1.949 |  |  |  |  |  |
|  |  | AnxAtt_Avg |  | 90.045 |  | 62 |  | 1.452 |  |  |  |  |  |
|  |  | AvoAtt_Avg |  | 86.659 |  | 62 |  | 1.398 |  |  |  |  |  |
|  |  | Obj_Avg |  | 16.525 |  | 62 |  | 0.267 |  |  |  |  |  |
|  | | | | | | | | | | | | | |

| **Table S2:** Between-groups comparisons (with adult-only attraction participants removed) | | | | | | | | | | | | | | | | | | | | | |
| --- | --- | --- | --- | --- | --- | --- | --- | --- | --- | --- | --- | --- | --- | --- | --- | --- | --- | --- | --- | --- | --- |
|  | | | | | | | | | | | | | | **95% Confidence Interval** | | | |  | | | |
|  | |  | | **Statistic** | | **df** | | **p** | | **Mean difference** | | **SE difference** | | **Lower** | | **Upper** | |  | | **Effect Size** | |
| SexFreq_Partner |  | Student's t |  | -1.026 |  | 182.000 |  | 0.306 |  | -0.672 |  | 0.654 |  | -1.963 |  | 0.620 |  | *d* |  | -0.156 |  |
| Proc_T |  | Student's t |  | 3.250 |  | 73.000 |  | 0.002 |  | 1.001 |  | 0.308 |  | 0.387 |  | 1.614 |  | *d* |  | 0.754 |  |
| Proc_A |  | Student's t |  | 3.556 |  | 73.000 |  | < .001 |  | 1.409 |  | 0.396 |  | 0.619 |  | 2.199 |  | *d* |  | 0.825 |  |
| Proc_B |  | Student's t |  | 3.473 |  | 73.000 |  | < .001 |  | 0.807 |  | 0.232 |  | 0.344 |  | 1.270 |  | *d* |  | 0.805 |  |
| Proc_E |  | Student's t |  | 2.448 |  | 73.000 |  | 0.017 |  | 0.968 |  | 0.395 |  | 0.180 |  | 1.756 |  | *d* |  | 0.568 |  |
| Sex_Esteem |  | Student's t |  | -0.375 |  | 178.000 |  | 0.708 |  | -0.052 |  | 0.139 |  | -0.327 |  | 0.223 |  | *d* |  | -0.058 |  |
| Sex_Preocc |  | Student's t |  | 0.024 |  | 181.000 |  | 0.981 |  | 0.003 |  | 0.131 |  | -0.256 |  | 0.262 |  | *d* |  | 0.004 |  |
| Sex_Dep |  | Student's t |  | 1.586 |  | 187.000 |  | 0.114 |  | 0.213 |  | 0.134 |  | -0.052 |  | 0.478 |  | *d* |  | 0.236 |  |
| Emo_Avg |  | Student's t |  | -1.167 |  | 191.000 |  | 0.245 |  | -0.207 |  | 0.177 |  | -0.556 |  | 0.143 |  | *d* |  | -0.172 |  |
| Schiz_Avg |  | Student's t |  | -0.420 |  | 178.000 |  | 0.675 |  | -0.035 |  | 0.084 |  | -0.201 |  | 0.131 |  | *d* |  | -0.064 |  |
| Bord_Avg |  | Student's t |  | 0.625 |  | 178.000 |  | 0.533 |  | 0.050 |  | 0.080 |  | -0.108 |  | 0.209 |  | *d* |  | 0.095 |  |
| NarPD_Avg |  | Student's t |  | -0.305 |  | 178.000 |  | 0.761 |  | -0.025 |  | 0.083 |  | -0.189 |  | 0.138 |  | *d* |  | -0.046 |  |
| AvPD_Avg |  | Student's t |  | -1.087 |  | 178.000 |  | 0.278 |  | -0.095 |  | 0.088 |  | -0.268 |  | 0.078 |  | *d* |  | -0.165 |  |
| OCPD_Avg |  | Student's t |  | -1.760 |  | 178.000 |  | 0.080 |  | -0.146 |  | 0.083 |  | -0.310 |  | 0.018 |  | *d* |  | -0.268 |  |
| Antisoc_Avg |  | Student's t |  | 0.032 |  | 178.000 |  | 0.974 |  | 0.003 |  | 0.091 |  | -0.177 |  | 0.183 |  | *d* |  | 0.005 |  |
| SecAtt_Avg |  | Student's t |  | 1.817 |  | 179.000 |  | 0.071 |  | 0.393 |  | 0.216 |  | -0.034 |  | 0.820 |  | *d* |  | 0.275 |  |
| AnxAtt_Avg |  | Student's t |  | 3.167 |  | 179.000 |  | 0.002 |  | 0.671 |  | 0.212 |  | 0.253 |  | 1.089 |  | *d* |  | 0.480 |  |
| AvoAtt_Avg |  | Student's t |  | -0.782 |  | 179.000 |  | 0.435 |  | -0.148 |  | 0.190 |  | -0.523 |  | 0.226 |  | *d* |  | -0.119 |  |
| Obj_Avg |  | Student's t |  | -1.191 |  | 172.000 |  | 0.235 |  | -0.094 |  | 0.079 |  | -0.251 |  | 0.062 |  | *d* |  | -0.184 |  |
|  | | | | | | | | | | | | | | | | | | | | | |
|  | | | | | | | | | | | | | | | | | | | | | |

| **Table S3:** Results from the binary logistic regression (with adult-only attraction participants removed) | | | | | | | | | | | |
| --- | --- | --- | --- | --- | --- | --- | --- | --- | --- | --- | --- |
|  |  |  |  |  |  |  |  |  |  |  |  |
| **Predictor** | | **Estimate** | | **SE** | | **Z** | | **p** | | **Odds ratio** | |
| Intercept |  | -12.763 |  | 9.030 |  | -1.413 |  | 0.158 |  | 0.000 |  |
| Age |  | 0.116 |  | 0.060 |  | 1.951 |  | 0.051 |  | 1.123 |  |
| Exclusivity |  | -3.003 |  | 1.857 |  | -1.617 |  | 0.106 |  | 0.050 |  |
| SexFreq_Partner |  | 0.031 |  | 0.125 |  | 0.251 |  | 0.802 |  | 1.032 |  |
| Sex_Esteem |  | 0.163 |  | 1.037 |  | 0.157 |  | 0.875 |  | 1.177 |  |
| Sex_Preocc |  | -3.846 |  | 1.900 |  | -2.024 |  | 0.043 |  | 0.021 |  |
| Sex_Dep |  | -0.202 |  | 1.270 |  | -0.159 |  | 0.874 |  | 0.817 |  |
| Emo_Avg |  | 2.452 |  | 1.551 |  | 1.581 |  | 0.114 |  | 11.610 |  |
| Schiz_Avg |  | 4.994 |  | 2.449 |  | 2.039 |  | 0.041 |  | 147.477 |  |
| Bord_Avg |  | 0.840 |  | 1.267 |  | 0.663 |  | 0.507 |  | 2.317 |  |
| NarPD_Avg |  | -2.093 |  | 1.530 |  | -1.368 |  | 0.171 |  | 0.123 |  |
| AvPD_Avg |  | 8.890 |  | 4.870 |  | 1.825 |  | 0.068 |  | 7255.447 |  |
| OCPD_Avg |  | -0.955 |  | 1.979 |  | -0.483 |  | 0.629 |  | 0.385 |  |
| Antisoc_Avg |  | -7.879 |  | 3.915 |  | -2.013 |  | 0.044 |  | 0.000 |  |
| SecAtt_Avg |  | -0.250 |  | 0.836 |  | -0.299 |  | 0.765 |  | 0.778 |  |
| AnxAtt_Avg |  | -2.332 |  | 1.261 |  | -1.850 |  | 0.064 |  | 0.097 |  |
| AvoAtt_Avg |  | 0.134 |  | 0.704 |  | 0.191 |  | 0.849 |  | 1.144 |  |
| Obj_Avg |  | 8.823 |  | 4.288 |  | 2.058 |  | 0.040 |  | 6790.000 |  |
| Proc_A |  | -4.576 |  | 1.981 |  | -2.310 |  | 0.021 |  | 0.010 |  |
| Proc_B |  | -2.713 |  | 1.513 |  | -1.793 |  | 0.073 |  | 0.066 |  |
| Proc_E |  | 4.105 |  | 1.887 |  | 2.176 |  | 0.030 |  | 60.631 |  |
| Note. Estimates represent the log odds of being a Doll Owner vs. Non-Owner | | | | | | | | | | | |
|  | | | | | | | | | | | |

| **Table S4a:** Contingency table for recoded self-reported offending (non-consensual sex) | | | | | | | | | |
| --- | --- | --- | --- | --- | --- | --- | --- | --- | --- |
|  | | | | **Group** | | | |  | |
| **Offending – Non-consensual sex** | |  | | **Non-Owner** | | **Doll Owner** | | **Total** | |
| No |  | Observed |  | 86 |  | 61 |  | 147 |  |
|  | | Expected |  | 84.840 |  | 62.160 |  | 147.000 |  |
| Yes |  | Observed |  | 15 |  | 13 |  | 28 |  |
|  | | Expected |  | 16.160 |  | 11.840 |  | 28.000 |  |
|  | | | | | | | | | |

| **Table S4b:** Contingency table for recoded self-reported offending (contact child abuse) | | | | | | | | | |
| --- | --- | --- | --- | --- | --- | --- | --- | --- | --- |
|  | | | | **Group** | | | |  | |
| **Offending – Non-consensual sex** | |  | | **Non-Owner** | | **Doll Owner** | | **Total** | |
| No |  | Observed |  | 81 |  | 56 |  | 137 |  |
|  | | Expected |  | 79.398 |  | 57.602 |  | 137.000 |  |
| Yes |  | Observed |  | 21 |  | 18 |  | 39 |  |
|  | | Expected |  | 22.602 |  | 16.398 |  | 39.000 |  |
|  | | | | | | | | | |

| **Table S4c:** Contingency table for recoded self-reported offending (indecent images) | | | | | | | | | |
| --- | --- | --- | --- | --- | --- | --- | --- | --- | --- |
|  | | | | **Group** | | | |  | |
| **Offending – Non-consensual sex** | |  | | **Non-Owner** | | **Doll Owner** | | **Total** | |
| No |  | Observed |  | 28 |  | 27 |  | 55 |  |
|  | | Expected |  | 31.743 |  | 23.257 |  | 55.000 |  |
| Yes |  | Observed |  | 73 |  | 47 |  | 120 |  |
|  | | Expected |  | 69.257 |  | 50.743 |  | 120.000 |  |
|  | | | | | | | | | |
